# Nationwide serological, molecular, and spatial assessment of Q fever in dromedary camels (*Camelus dromedarius*) in Jordan: A One Health perspective

**DOI:** 10.14202/vetworld.2026.678-692

**Published:** 2026-02-23

**Authors:** Ruba Alomari, Majid Hawawsheh, Shahin Baiomy, Nacira Ramdani

**Affiliations:** 1 Al-Khanasiri Department for Livestock and Rangeland Research, National Agricultural Research Center (NARC), Al-Baqa’a, Jordan; 2 Ministry of Agriculture, Amman, Jordan; 3 European Commission for the Control of Foot-and-Mouth Disease, Food and Agriculture Organization of the United Nations, Rome, Italy; 4 Regional Veterinary Laboratory of El Oued, National Institute of Veterinary Medicine, El Oued, Algeria; 5 Management of Animal Health and Productions Laboratory, Institute of Veterinary Sciences, University of Frères Mentouri Constantine 1, Constantine, Algeria

**Keywords:** camel epidemiology, *Camelus dromedarius*, *Coxiella burnetii*, Jordan, molecular detection, Q fever, risk factors, spatial distribution

## Abstract

**Background and Aim:**

Q fever, caused by Coxiella burnetii, is a globally distributed zoonosis with major public health and livestock production implications. Dromedary camels (*Camelus dromedarius*) are increasingly recognized as important reservoirs, particularly in arid and semi-arid regions. In Jordan, however, comprehensive national-level data integrating serology, molecular detection, and spatial epidemiology are lacking. This study aimed to estimate the serological and molecular prevalence of Q fever in camels, identify associated risk factors, and describe the spatial distribution of infection across Jordan.

**Materials and Methods:**

A nationwide cross-sectional study was conducted between July and October 2022 using a two-stage cluster sampling design. A total of 468 camels from 31 villages across all camel-rearing governorates were sampled. Serum samples were tested for anti-*C. burnetii* antibodies using a commercial indirect multi-species enzyme-linked immunosorbent assay, while whole blood was examined for *C. burnetii* DNA using conventional polymerase chain reaction (PCR) assays targeting IS1111 and CB-1 genes. Survey-weighted prevalence estimates were calculated, and risk factors were evaluated using univariate and multivariate survey-weighted logistic regression. Spatial distribution was mapped at the governorate level.

**Results:**

The weighted prevalence of Q fever was 88.75% (95% confidence interval: 79.26%–98.23%) using parallel interpretation of serological and molecular tests. Individually, prevalence was 44.02% by enzyme-linked immunosorbent assay and 68.93% by PCR. Significant risk factors included age ≥3 years, contact with other camel herds, Sofor breed, light or absent tick infestation, and improper disposal of abortion materials. Co-herding with other species and a history of abortion were associated with reduced odds of positivity. Marked spatial heterogeneity was observed, with the highest prevalence in Tafilah governorate and the lowest in Zarqa.

**Conclusion:**

This first nationwide, integrated serological, molecular, and spatial study demonstrates an exceptionally high burden of Q fever in Jordanian camels, highlighting their critical role as a reservoir. The findings underscore the urgent need to incorporate camels into national Q fever surveillance and control programs within a coordinated One Health framework involving animal, human, and environmental health sectors.

## INTRODUCTION

The obligate intracellular bacterium *Coxiella burnetii* is the causative agent of Q fever, a globally emerging zoonotic disease that affects a wide range of hosts, including humans, domestic ruminants, wildlife, and ticks [1-6]. Although ticks and ruminants have been identified as the principal reservoirs during outbreaks, the role of ticks remains unclear and warrants further investigation. There is ongoing debate as to whether ticks primarily act as biological vectors facilitating bacterial transmission or function mainly as environmental reservoirs that maintain bacterial circulation [6, 7]. In livestock, infection with *C. burnetii* is associated with major reproductive disorders, including abortion and infertility, resulting in substantial economic losses [7]. The World Health Organization (WHO) recognizes Q fever as a high-priority zoonosis in humans. Infection is predominantly acquired through the inhalation of contaminated aerosols and typically manifests as an acute febrile illness that may progress to severe chronic outcomes, such as endocarditis, thereby posing a significant occupational risk to farmers, veterinarians, slaughterhouse workers, and other individuals with close animal contact [8–111].

Despite its recognized importance, the true prevalence of Q fever remains poorly defined, largely due to frequent misdiagnosis in both animals and humans. Recent meta-analyses have reported a global herd-level seroprevalence of 44.4% in cattle, highlighting the widespread and ubiquitous nature of *C. burnetii* infection [12]. This burden is particularly pronounced in the Middle East. In Saudi Arabia, herd-level seroprevalence exceeds 92% in goats and 80% in sheep [13]. Similarly, high seroprevalence rates have been documented in sheep (up to 50%) and goats (12%–51.4%) across various regions of Egypt [9, 14–17]. These elevated infection rates in livestock are mirrored by substantial human seroprevalence levels of 35%, 52%, and 24.2% in Saudi Arabia, Egypt, and Jordan, respectively [9, 10, 18].

The role of camels as a key reservoir for Q fever has become increasingly evident, contributing to shifts in regional epidemiology, particularly in the Middle East [4, 19, 20]. Alarmingly high seroprevalence levels have been reported in neighboring countries, ranging from 66% in Egypt to 100% in eastern Ethiopia [20, 21]. In Saudi Arabia, camels exhibit a herd-level seropositivity of 92.9% [10]. The high prevalence of infection, combined with intensive camel husbandry systems and common cultural practices such as the consumption of raw camel milk, has positioned camels as a potent amplifier of human infection risk in recent years [20, 23]. Nevertheless, the precise role of camels in the zoonotic transmission cycle of *C. burnetii* remains insufficiently investigated, representing a significant epidemiological knowledge gap.

In Jordan, available data on Q fever are limited. A previous study reported a seroprevalence of 49.6% in camels in southern Jordan and an overall prevalence of 63% in ruminants nationwide; however, these investigations were geographically restricted and lacked molecular confirmation and spatial analysis [16, 23].

Despite growing recognition of Q fever as a major zoonotic threat and the emerging importance of camels as reservoirs, critical knowledge gaps persist regarding the epidemiology of *C. burnetii* infection in *C. dromedarius*, particularly in Jordan. Existing studies are limited in geographic scope and are largely confined to serological evidence, without integrating molecular confirmation or spatial analysis. Consequently, the relative contribution of active infection versus past exposure remains unclear, and the influence of animal-level, herd-level, and management-related risk factors has not been comprehensively quantified at the national-level. Moreover, the absence of survey-weighted analytical approaches limits the generalizability of available estimates. This lack of integrated serological, molecular, and spatial data hinders accurate risk assessment and constrains the development of evidence-based surveillance and control strategies within a One Health framework.

To address these gaps, the present study aimed to conduct a nationwide, representative assessment of Q fever in *C. dromedarius* in Jordan by integrating serological, molecular, and spatial epidemiological approaches. Specifically, the study sought to estimate the seroprevalence and molecular prevalence of *C. burnetii* using enzyme-linked immunosorbent assay and polymerase chain reaction (PCR), identify animal- and management-related risk factors associated with infection through survey-weighted regression modeling, and map the spatial distribution of Q fever across camel-rearing governorates. By generating robust, population-representative data, this study aims to clarify the epidemiological role of camels in Q fever transmission and provide a scientific basis for strengthening national surveillance and targeted control measures aligned with One Health priorities.

## MATERIALS AND METHODS

### Ethical approval

The Scientific Research Committee of the Scientific Research Support Fund (SRSF) approved this study, including the ethical consideration and animal sampling acceptance letter (AGR/1/17/2021). The camel owners cooperated with the vet team and were granted permission to collect samples from their herds. The vet team discussed with the owners the importance of Q fever and the objective of the study. It was emphasized to all of them that their enrollment is completely voluntary and that data were collected and stored confidentially. Animal blood collection was performed aseptically under the supervision of a veterinarian, following the relevant animal welfare guidelines. All procedures in this study complied with the guidelines of the Animal Research Reporting of *In Vivo* Experiments 2.0 [24].

### Study period and location

This study was conducted from July to October 2022 in Jordan, officially known as the Hashemite Kingdom of Jordan. Jordan is a Middle Eastern country located at the crossroads of Europe, Africa, and Asia. Jordan is divided into 12 governorates. The country’s topography is diverse: The southern region is dominated by desert with an arid to semi-arid climate, whereas the northern highlands are relatively more temperate. Overall, most of Jordan’s land area is classified as desert. Winters can be cold in some regions, whereas summers are typically hot and dry. Rainfall varies widely, from as little as 30 mm in arid zones to approximately 572 mm in the hilly northwest, with most precipitation occurring between October and May.

**Figure 1:**
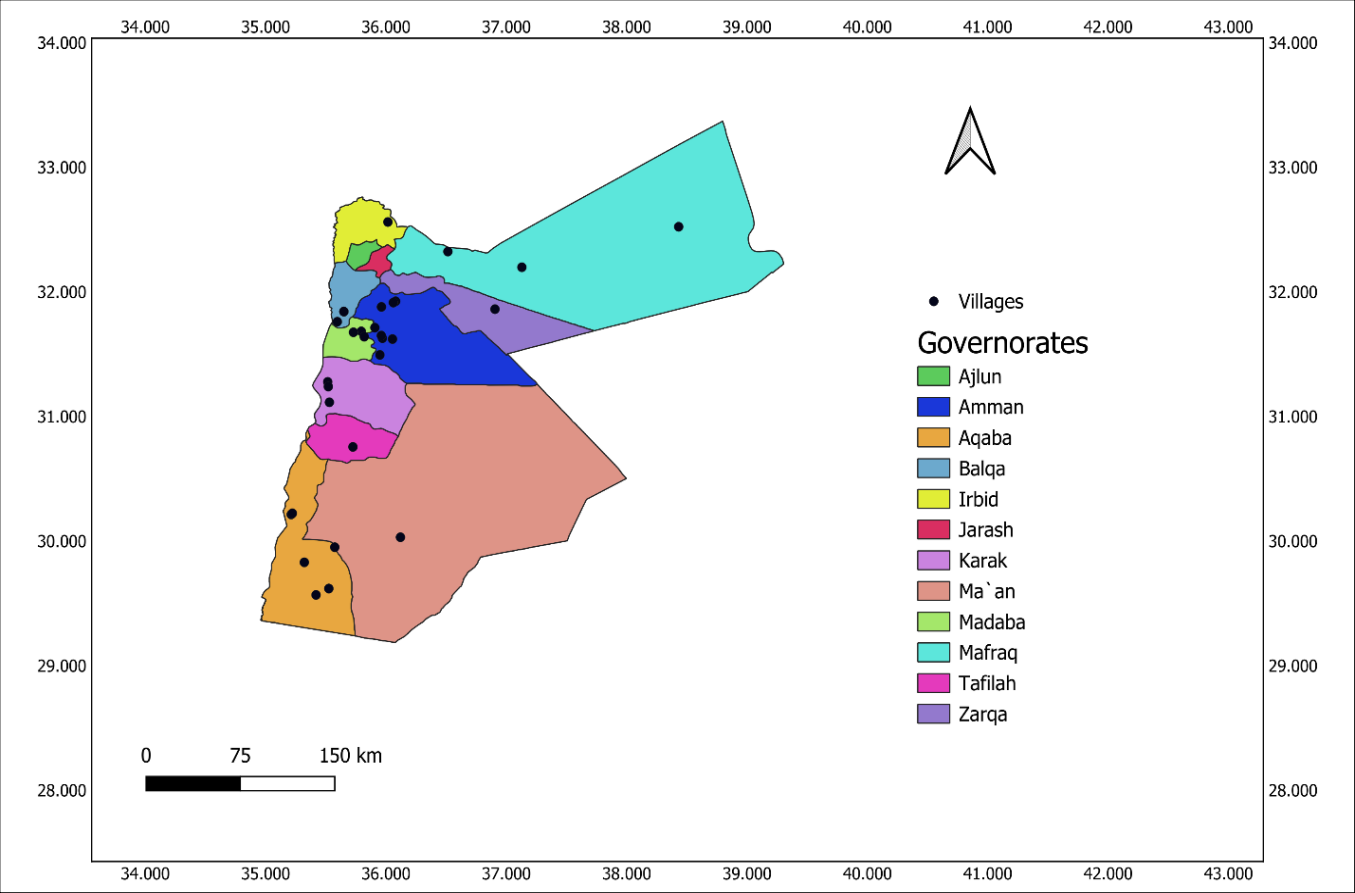
Map showing the localization of sampled villages and governorates in Jordan where dromedary camels were surveyed for Q fever.

Jordan’s climate and diverse topography, including desert and highlands, make it a good place to raise and sustain camel populations, especially in arid and semi-arid areas. Thus, it may affect camel breeding and the possible spread of Q fever. The camel population was distributed across 10 of 12 Jordanian governorates, with herds typically clustered within villages. According to the Ministry of Agriculture, there are an estimated 14,250 camels in the country. Figure 1 shows the map of the study area.

### Calculation of sample size

A cross-sectional study was conducted between July and October 2022 using a two-stage cluster sampling design.

The initial sample size (n) was calculated using Thrusfield’s formula [25, 26]:

n = ((1.96)^2^ × p(1–p))/d^2^

where: p = expected seroprevalence (49.6%), as calculated from a previous study in southern Jordan [23]. d = allowable error (0.05). 1.96 = Z-value for 95% confidence level (CI).

Based on this formula, the initial sample size was estimated to be 231.

To account for clustering in the sampling design, the final sample size (N) was adjusted using the design effect (D) as described by Bennett *et al*. [27]:

N = n × D

D = 1 + (b–1)ρ

where: ρ (roh) = intra-cluster correlation coefficient (ICC) (0.07). b = average number of camels sampled per household (15).

Applying this adjustment resulted in a minimum sample size of 462.

The assumed ICC of 0.07 reflects moderate clustering at the village level, which aligns with reported ranges for infectious diseases in livestock populations (typically 0.01–0.20) [25].

Based on the list of owners’ names and the number of camels in their farms obtained from the Ministry of Agriculture. The sample size was determined to be 15 camels per village based on the average herd size (15 camels). The herd that had fewer camels was excluded from the list. Therefore, the number of clusters was determined by dividing the adjusted sample size (462) by the fixed sample size of 15, yielding 31 clusters (villages). The herds from the villages were randomly selected from the list of owners’ names, with no more than 5 samples per owner to avoid owner compliance. The exclusion criteria included camels younger than 6 months of age, as the enzyme-linked immunosorbent assay (ELISA) test may detect maternal antibodies, as previously reported [23].

### Structure of the questionnaire and data collection

Camel samples and data were collected between July 2022 and October 2022. A predesigned questionnaire was used to collect data through personal interviews. A team, in collaboration with a local vet from the Ministry of Agriculture in each village, interviewed the workers and owners face-to-face, using simple language and building on the good relationship and trust between them, to ensure full and accurate data.

The questionnaire covers various factors at the animal and herd levels, including location (governorate and village), animal characteristics (age, species, origin, and color), the husbandry system practiced, herd disease history, and health practices. In addition to collecting data on abortion history, the presence of sheep and goats on the farm, the presence of ticks (light (15–60), mild (61–200), and severe ≥200), and the control method used were also recorded. Owners were also interviewed about their general knowledge of the clinical signs of Q fever in animals and about the application of sanitary practices for the removal of birth products and for the consumption of raw milk.

### Blood sample collection

Following strict biosafety measures and wearing personal protective equipment, a total of 8–10 mL of blood was collected from the jugular vein of 469 camels, with preference given to those infested with ticks. Both the plain and ethylenediaminetetraacetic acid (EDTA) tubes were carefully labeled with the corresponding governorate, village, and herd. The samples were placed in plain thrombin-containing vacutainer tubes (Catalog No. VP20021S, BD Diagnostic, Oxford, UK), allowed to clot for 30 min, and then centrifuged at 5,000 × *g* for 5 min. Serum was collected in two Eppendorf tubes labeled with a specific tracking number (Catalog No. 0030123328, Sigma-Aldrich, Missouri, United States). Both serum and EDTA samples were transported within 24 h at 4°C in ice boxes to maintain integrity until arrival at the Al-Khanasri Department for Livestock and Rangeland Research (Mafraq, Jordan)/National Agricultural Research Center (NARC), upon arrival, samples were stored at −20°C until laboratory analysis.

### Laboratory diagnosis

#### Serological test (Q fever ELISA)

The Q fever antibody indirect multi-species ELISA kit (IDVet Innovative Diagnostics-MS ver1117, France) was used to detect *C. burnetii* antibodies in all serum samples, following the manufacturer’s instructions. The diagnostic test sensitivity and specificity of the kit’s manufacturer were 100% (95% CI: 89.28%–100%) and 100% (95% CI: 97.75%–100%), respectively. In addition to the tested samples, positive and negative control sera were included in each plate in duplicate; the optical density (OD) readings for the plate were obtained at 450 nm on the ELISA reader (Multiscan FC, Thermo Scientific, USA) after adding the stop solution. The laboratory staff tested the samples as blind samples with a unique lab number.

The test is valid when the OD of the PC is greater than 0.350 and the ratio of the PC to the NC is greater than 3 (OD PC >0.350 & OD PC /OD NC >3). The S/P% was calculated according to the manufacturer’s formula, where OD is the mean of the two duplicate samples or controls, measured at 450 nm.

S/P % = (OD sample - OD NC / OD PC – OD NC) x 100

where N and P are the negative and positive OD controls, respectively. The manufacturer’s recommended cut-offs were used to interpret the ELISA results, with a sample-to-positive ratio (S/P%) of >50% considered positive. Sera were seropositive if the S/P % was> 50% and ≤80%, strong positive if the S/P % was≥80%, and negative if the S/P % was ≤40%. However, all the inconclusive samples were considered negative. Samples that returned an equivocal S/P% of 40% to ≤50% were repeated (n=10). A sample that crossed the positive cutoff threshold on the repeat test was reclassified as positive; otherwise, it was counted as negative for subsequent analyses.

#### DNA extraction from the EDTA samples

Whole blood samples in EDTA tubes were used for DNA extraction. DNA was extracted using the Wizard Genomic DNA Purification Kit (Promega, USA). The extraction procedure was performed in a dedicated extraction room, using equipment and disposables certified for PCR use to prevent contamination. The quality of the extracted DNA was evaluated by electrophoresis on agarose 1% agarose (Promega) in 1X TAE buffer with 7 μl Red Safe Nucleic Acid staining solution (20,000X) (Intron Biotechnology, JH science, NJ) for 45 min at 120 V compared to 100 bp Ladder RTU (GeneDirex, USA).

#### Conventional PCR for the detection of Q fever

Detection of *C. burnetii* DNA was performed using two distinct conventional PCR assays. The first targeted a 257-bp fragment of the superoxide dismutase enzyme gene using primers C.B.1 (5´-ACTCAACGTACTGGAACCGC-3´) and C.B.-2 (5´-TAGCTGAAGCCAATT CGCC-3´) [28]. The second targeted a 687-bp fragment within the repetitive, transposon-like IS1111 region using primers Trans 1 (5´-TGGTATTCTTGCCGATGAC-3´) and Trans 2 (5´-GATCGTAACTGCTTAATAAACCG-3´) [29]. The oligos were obtained from the Macrogen company (Korea).

The PCR reaction was performed in a final volume of 20 μL. The PCR mixture contained 4 μL of 5× HOT FIREPol® Blend Master Mix (Solis BioDyne, Tartu, Estonia), 1 μL of 10 μM of each respective primer, 2 μL of template DNA, and nuclease-free water to adjust the final volume. The reaction was performed in a separate room from that of extraction and under laminar flow (Msc-ADVANTAGE, Thermo Scientific, Germany) using materials and disposables certified for PCR. Negative controls (reaction without DNA template) were included in each reaction, while *C. burnetii* DNA from a confirmed positive laboratory sample was used as a positive control. Amplification was performed in a ProFlex PCR system thermocycler (Applied Biosystems, Foster City, CA, USA) under the following conditions: initial denaturation at 95°C for 15 min, followed by 35 cycles of 95°C for 45 s, 61°C for 45 s for CB1 primer, 56°C for 45 s for Trans primer, and 72°C for 2 min. The final extension step was performed at 72°C for 10 min.

The PCR products were analyzed via electrophoresis on a 2% (w/v) agarose gel (Promega) prepared in 1X TAE buffer. Gels were run at 120 V for 45 min, stained with 7 μl of Red Safe Nucleic Acid staining solution (20,000X) (Intron Biotechnology, JH Science, NJ, USA), and visualized under ultraviolet transillumination. The molecular weight of the obtained product was determined using the 100 bp DNA Ladder RTU (GeneDirex) as a molecular weight marker. When an amplicon of the expected size was detected, the sample was considered positive.

### Statistical analysis

#### Data management and validation

Data collection forms, completed by field veterinarians, were entered into Excel spreadsheets (Microsoft Office 365, Washington, USA) and subsequently cleaned and coded for statistical analysis. The data were first checked by the person responsible for data entry and then independently reviewed by the data analyst who conducted the statistical analyses. Missing values were removed before analysis.

#### Weighted prevalence estimation

Survey-weighted prevalence estimates were calculated using the survey package (version 3.29) in R to obtain accurate and generalizable estimates of Q fever prevalence in camels [30]. A complex survey design was specified using the svydesign() function, where the village was the primary sampling unit (PSU), and sampling weights were applied via the weight variable. This adjustment corrects for over- or under-representation of animals from certain clusters and ensures that the target population is represented in the prevalence estimates.

Sampling weights were calculated using the following formula to adjust for unequal probabilities of animal selection within villages:


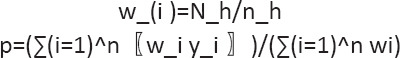


Where: Wi= weight i. N_his the total number of camels in village h. n_h is the number of camels sampled from the village. i= individual camel in village. y i= disease status (0/1). p = weighted prevalence

This weight is applied to each sampled camel to ensure that the prevalence estimates reflect the broader population structure across all villages.

The svymean() function was then used to estimate the weighted prevalence of Q fever based on different diagnostic outcomes, including parallel and series interpretation of both ELISA and PCR results, as well as individual ELISA and PCR results. CI for these prevalence estimates were computed using confint(), which accounts for the complex survey design and provides robust variance estimates. This method ensures unbiased estimates of Q fever seroprevalence and molecular prevalence in the camel population sampled, accommodating both clustering and weighting in the survey structure.

#### Risk factor analysis (RFA)

A univariable survey-weighted logistic regression was first conducted for each potential explanatory variable to investigate the risk factors associated with Q fever positivity among camels using the svyglm() function from the survey package in R [30], accounting for clustering and sampling weights. The dependent variable was the Q fever infection status based on a parallel testing approach.

Therefore, to reduce the risk of misclassification and increase diagnostic confidence, we adopted a combined interpretation of the two tests, which represents a more reliable approach for accurately assessing Q fever status in camels.

The following variables were included in the univariable analysis: age category, sex, husbandry system, presence of ticks on camels, location of tick infestation, abortion history, camel breed, co-herding status, animal origin (purchased vs. homebred), production purpose (e.g., milk, meat), abortion management practices, contact with other herds, presence of dogs, presence of cattle, presence of sheep, presence of goats, and tick density in camels. Variables with a p-value ≤ 0.25 in the univariate analysis were retained for inclusion in the multivariate logistic regression model [31]. The potential multicollinearity was computed using the Cramér’s V coefficient. Variables were considered strongly correlated if the coefficient was >0.4 [32], and only the most biologically pertinent variables for Q fever were retained. The final model, estimated using svyglm(), was derived through a manual backward stepwise procedure guided by the global Wald test using a quasibinomial family (a binomial model with an added dispersion parameter to account for overdispersion due to extra variability in clustered survey data). Potential confounders were identified using a change-in-estimate criterion: a variable was considered a confounder if its inclusion or exclusion altered the main exposure’s regression coefficient by ≥20%. Non-significant predictors (p > 0.05) were sequentially removed during model building, but variables identified as potential confounders were retained regardless of statistical significance to ensure the stability and validity of the model.

The model controlled for all eligible predictors and accounted for the complex survey design, providing unbiased estimates and valid inference. All analyses and visualizations were conducted using R (version 4.5.0) [33]. Model diagnostics were performed to assess the performance of the model. A global Wald test was conducted to assess the joint significance of the predictors. The model fit was further examined using the fit.svyglm function from the poliscidata package [34], which provided pseudo and adjusted R-squared values. Model calibration was assessed with the Hosmer–Lemeshow test, while overall goodness-of-fit was evaluated using Pearson’s Chi-squared test with Rao–Scott second-order correction to account for the complex survey design.

#### Spatial analysis

A thematic choropleth map (prevalence and sample size per governorate) and a reference location map (villages and governorates) were produced using QGIS (Version 3.44.4 “Solothurn”) with the following coordinate reference system: EPSG:4326- WGS 84 [35]. The weighted prevalence data of parallel testing and sample size were integrated into a shapefile of Jordan’s administrative boundaries (GADM). The weighted prevalence per governorate was calculated using the following formula:

Weighted prevalence=(Σ(W_(i.) X_i))/(ΣW_i)

where X_i is the test result (1 = positive, 0 = negative) and W_i is the sampling weight for each observation. The following equation is expressed as follows: This approach accounts for unequal selection probabilities and provides more representative estimates across regions.

#### Sensitivity analysis

Several sensitivity checks were performed to evaluate the robustness of the risk factor analysis. Collinear variables were assessed using Cramér’s V coefficient, and alternative model specifications were tested by replacing or removing collinear predictors to compare stability across models. Potential confounding factors were systematically assessed, and multiple interaction terms between explanatory variables were explored. Both backward and forward stepwise selection procedures were conducted to ensure that the final model retained significant predictors while maintaining good model fit. These approaches confirmed the stability and robustness of the final survey-weighted logistic regression model.

### Limitations of the methods

An alternative analytical strategy would have been to use a generalized linear mixed model with village as a random effect, treating individual infection status as the dependent variable. However, this approach does not accommodate sampling weights and therefore would not provide representative weighted prevalence or risk factor estimates for the target population. Instead, we specified a complex survey design in R using the survey package, with villages as the PSU through the svydesign() function and applied sampling weights in prevalence estimation via svymean(). Although this framework ensures unbiased, population-representative estimates, it does not explicitly model random variability between villages, which should be acknowledged as a limitation.

In addition to the statistical limitations mentioned above, the cross-sectional design of this study imposes several methodological restrictions. The analysis relied on serological data, precluding the direct assessment of current infection. Biological samples indicative of active shedding or abortive events, such as milk, vaginal swabs, or placental tissues, were not collected, restricting our capacity to detect intermittent shedding or relate seropositivity to abortion. In addition, young camels (≤1 year old) have been excluded from sampling, as maternal antibodies may interfere with antibody detection. Thus, this exclusion might affect the representativeness of the seroprevalence results. Additionally, using acaricides for tick control may lead to misclassification of tick infestation criteria and, in turn, misleading seropositivity in light infestations.

## RESULTS

### Survey-weighted prevalence estimates

The weighted prevalence of Q fever in camels, adjusted for unequal sampling within villages, varied depending on the diagnostic method used. Using the parallel testing approach (positive if either ELISA or PCR was positive), the prevalence was 88.75% (95% CI: 79.26%–98.23%). In contrast, the series testing approach (positive only if both ELISA and PCR were positive) yielded a lower prevalence of 24.21% (95% CI: 8.86%–39.55%). When individual diagnostic tests were considered, the prevalence was 44.02% (95% CI: 17.68%–70.36%) based on ELISA and 68.93% (95% CI: 47.48%–90.38%) based on PCR analysis of blood samples (Table 1).

**Table 1:** Cross-tabulation of ELISA and PCR results for *Coxiella burnetii* in dromedary camels (n = 468).

ELISA	PCR –ve	PCR +ve	Total
–ve	92	118	210
+ve	115	143	258
Total	207	261	468

–ve = Negative, +ve = Positive, ELISA = Enzyme-linked immunosorbent assay, PCR = Polymerase chain reaction

### Univariable risk factor screening

A univariable survey-weighted logistic regression analysis was performed to screen potential predictors associated with Q fever seropositivity in camels (Table 2). Variables with a p ≤ 0.25 were considered eligible for inclusion in multivariable modeling. These variables included age, gender, husbandry system, tick presence, tick density, places of tick infestation, abortion history, breed, co-herding status, animal origin, production purpose, abortion management practices, contact with other herds, and presence of sheep. Due to multicollinearity, the variables presence of sheep, places of tick infestation, tick presence, and gender were excluded from further analysis.

**Table 2:** Univariate analysis of animal-related factors potentially associated with Q fever seropositivity and molecular results in Jordanian camels.

Variable	Category	Category percentage	Weighted prevalence (%)	p-value
Age	1 year	10.90%	74.41	0.05**
	2–3 years	0.85%	90.26	
	≥3 years	88.25%	90.83	
Gender	Male	7.26%	63.84	<0.001**
	Female	92.74%	90.53	
Husbandry System	Stable	32.91%	95.05	0.31
	Pasture	57.48%	83.06	
	Mixed	9.62%	87.55	
Tick’s presence	No	30.77%	95.5	0.03**
	Yes	69.23%	81.94	
Places of tick infestation	No presence	30.77%	95.5	<0.01**
	Udder or Testes, Sternum	0.21%	100	
	Udder or testes, inguinal, perineum, and sternum	0.85%	50	
	Udder or Testes, Perineum	0.64%	100	
	Udder or Testes, Perineum, and Sternum	4.27%	89.58	
	Sternum	19.02%	79.2	
	Inguinal	0.64%	33.33	
	Inguinal, Sternum	0.21%	0	
	Inguinal, perineum, and sternum	0.43%	50	
	Perineum	18.80%	91.89	
	Perineum, Sternum	21.79%	76.93	
	Perineum, Sternum, and Tail	0.43%	0	
	Perineum, Chest	0.21%	0	
	Perineum, chest, and sternum	0.64%	100	
	Chest	1.07%	100	
Abortion history	Yes	1.07%	60	<0.01**
	No	98.93%	88.84	
Breed	Waddah	13.25%	67.77	0.18*
	Shageh	20.30%	88.53	
	Majaheem	15.81%	94.11	
	Sofor	28.21%	89.8	
	Shaele and Homor	22.44%	94.52	
Co_herding status	No	64.32%	91.39	0.03**
	Yes	35.68%	73.18	
Animal origin	Born on a farm	98.93%	88.77	0.17*
	Bought	1.07%	80	
Production purpose	Milk, Meat	89.32%	87.86	<0.001**
	Milk, meat, and racing	8.55%	93.79	
	Milk, Meat, and Prestige	2.14%	20	
Abortion management	Waste container	55.98%	30	<0.001**
	No abortion	34.62%	90.22	
	Thrown in the valley	7.26%	85.44	
	Burning or burial	2.14%	96.42	
Contact with other herds	No	83.12%	83.83	0.09*
	Yes	16.88%	96.03	
The presence of dogs	No	69.87%	88.65	0.93
	Yes	30.13%	89.24	
Presence of sheep	No	49.57%	92.38	0.16*
	Yes	50.43%	78.6	
Goat	No	66.45%	88.84	0.93
	Yes	33.55%	88.26	
Tick’s Density	No presence	50.21%	95.5	0.04**
	Light (15–60)	16.45%	88.05	
	Mild (61–200)	30.77%	57.31	
	Severe ≥200	2.56%	59.53	
Herd size	Small	33.05%	77.32	0.06*
	Medium	48.19%	89.2	
	Large	18.76%	94.81	
Cattle presence	No	100%	88.75	–

–ve = Negative, +ve = Positive,

** = Variables significantly associated with Q fever seropositivity,

* = Variables with a p-value ≤ 0.25 to be included in the multivariate analysis

### Multivariable risk factor analysis

The final multivariable survey-weighted logistic regression model identified several significant predictors of Q fever positivity (Table 3). Camels aged ≥3 years had significantly higher odds of positivity compared with those aged 1 year (p = 0.001; OR = 4.02, 95% CI: 2.00–8.07). Tick infestation status was a strong predictor of infection. Camels without ticks showed markedly higher odds of positivity (p = 0.009; OR = 11.57, 95% CI: 2.33–57.53), followed by camels with light tick infestation (15–60 ticks) (p = 0.015; OR = 6.78, 95% CI: 1.73–26.55), when compared with animals with moderate tick burden (61–200 ticks).

**Table 3:** Multivariate analysis of factors associated with seropositivity and molecular results of Q fever in camels in Jordan.

Predictor	Category	Log Odds	OR	95% CI (OR)	p-value
Age	≥3 years	1.39	4.02	(2.00–8.07)	0.001
Tick Density	No ticks	2.45	11.57	(2.33–57.53)	0.009
	Light (15–60)	1.91	6.78	(1.73–26.55)	0.015
Breed	Sofor	1.76	5.81	(1.49–22.58)	0.023
Co-herding with other species	Yes	–1.08	0.34	(0.16–0.72)	0.013
Contact with other herds	Yes	1.79	6.03	(1.56–23.25)	0.02
Abortion Management	Burn/Bury	3.76	43.13	(3.68–505.99)	0.009
	Thrown in the valley	1.71	5.5	(1.60–18.88)	0.016
Abortion History	No	–1.76	0.17	(0.03–0.85)	0.049

OR = Odds ratio, CI = Confidence interval

Breed was also significantly associated with Q fever seropositivity. Camels of the Sofor breed had higher odds of infection than those of the Waddah breed (p = 0.023; OR = 5.81, 95% CI: 1.49–22.58). In contrast, camels that were co-herded with other animal species were significantly less likely to test positive, exhibiting a 66% reduction in the odds of infection (p = 0.013; OR = 0.34, 95% CI: 0.16–0.72). However, camels that had contact with other herds were at substantially greater risk, with approximately a five-fold increase in the odds of seropositivity (*p* = 0.020; OR = 6.03, 95% CI: 1.56–23.25).

### Abortion history and management practices

Abortion management practices were strongly associated with Q fever seropositivity. Disposal of aborted materials by burial or burning was associated with markedly increased odds of seropositivity (p = 0.009; OR = 43.13, 95% CI: 3.68–505.99), while disposal by dumping in valleys also showed an elevated risk (p = 0.016; OR = 5.50, 95% CI: 1.60–18.88), compared with disposal in waste containers. Conversely, animals with a history of abortion had significantly lower odds of testing positive (OR = 0.17, 95% CI: 0.03–0.85; p = 0.049), suggesting a potential protective effect.

### Model performance and diagnostics

The final model demonstrated strong overall statistical significance. The global Wald test confirmed that age, breed, tick density, co-herding status, contact with other herds, abortion management practices, and abortion history were jointly significant predictors of outcome variation (p < 0.001). The pseudo-R-squared value was 0.287, indicating moderate explanatory power. The Hosmer–Lemeshow test yielded a chi-squared statistic of 5.66 on 8 degrees of freedom (p = 0.69), indicating good model calibration. Additionally, Pearson’s Chi-squared test with Rao–Scott correction revealed a significant association between observed and expected frequencies (F = 18.54, p < 0.001), confirming that the model adequately captured the data structure while accounting for the complex survey design.

### Spatial distribution of seroprevalence

**Figure 2:**
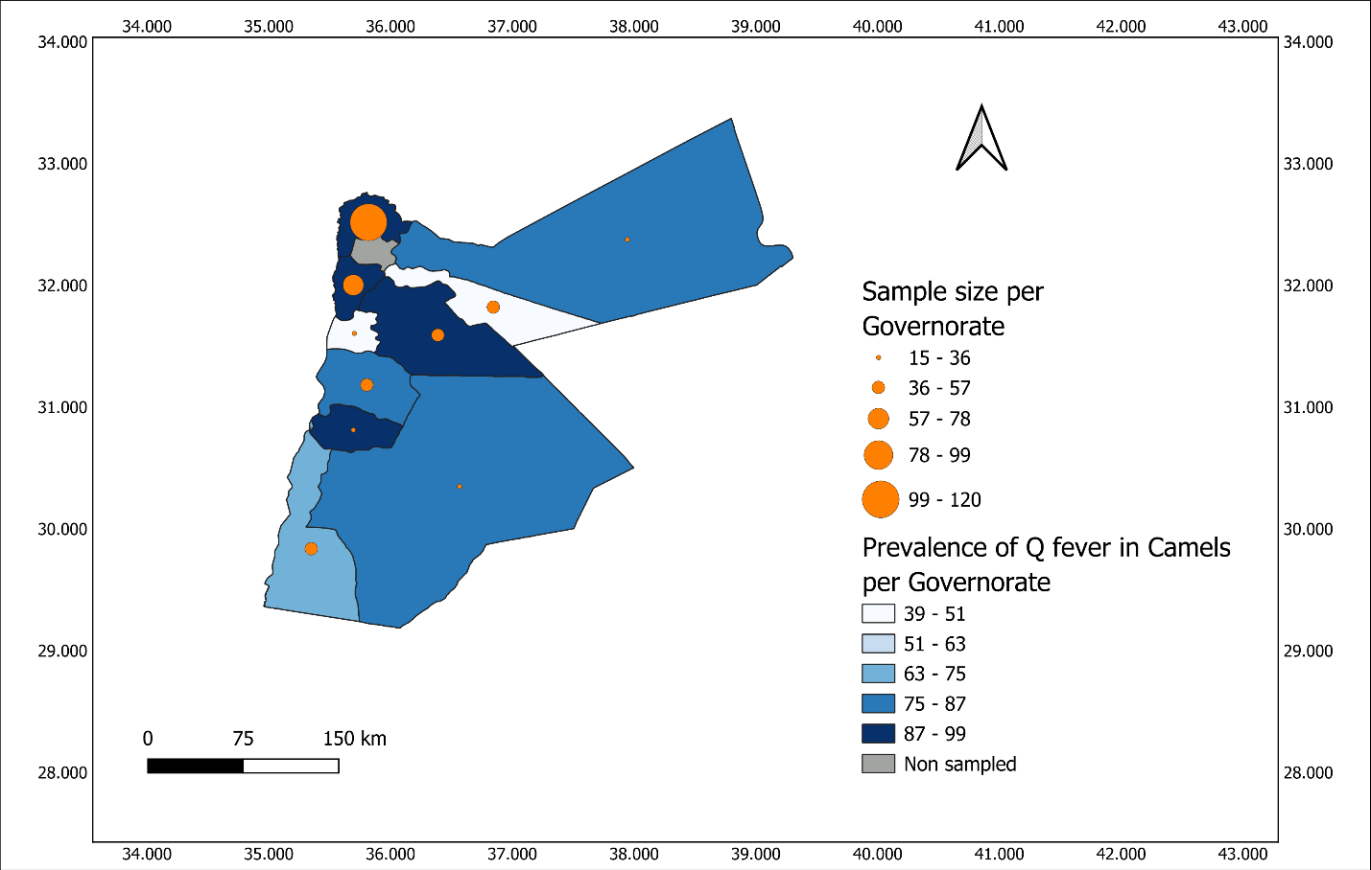
Geographic distribution of sample size per governorate (proportional symbols) and weighted prevalence of Q fever in dromedary camels across Jordanian governorates.

The weighted seroprevalence of Q fever among camels showed marked geographic variation across Jordanian governorates. The highest prevalence was observed in Tafilah (98.74%, CI: 96.93–100.56), followed by Irbid (93.31%, CI: 88.86–97.77), Balqa (92.78%, CI: 86.69–98.86), and Amman (92.48%, CI: 84.31–100.64). Moderate prevalence levels were recorded in Karak (84.00%, CI: 74.12–93.88), Ma’an (83.33%, CI: 68.41–98.26), and Mafraq (85.71%, CI: 67.36–104.06). In contrast, lower prevalence was reported in Aqaba (69.40%, CI: 56.10–82.70), Madaba (40.00%, CI: 15.18–64.82), and Zarqa (38.61%, CI: 23.48–53.73) (Figure 2).

## DISCUSSION

### Widespread *C. burnetii* infection and active circulation in camel herds

To the best of our knowledge, this is the first comprehensive investigation of *C. burnetii* infection in camel herds in Jordan. The findings demonstrate a substantial prevalence of both *C. burnetii*–specific antibodies (seroprevalence, 44.02%; 95% CI: 17.68%–70.36%) and bacterial DNA (molecular prevalence, 68.93%; 95% CI: 47.48%–90.38%), indicating not only widespread previous exposure but also active infection within camel herds. These results strongly support the epidemiological significance of camels as a reservoir in local Q fever transmission. The nationwide seroprevalence estimate falls within the broad range (15.8%–80%) reported across the Middle East and North Africa [4, 23, 36, 37]. The difference between our findings and the 49.6% seroprevalence reported previously in southern Jordan [23] is likely attributable to differences in sampling design and broader geographic coverage, highlighting the heterogeneous nature of *C. burnetii* transmission and the importance of camels as maintenance hosts within Jordan’s pastoral and mixed-livestock systems.

Previous studies have emphasized the interaction between genetic susceptibility in camels and the complexity of risk and protective factors across different ecological contexts [1, 38]. This interaction may explain divergence in identified risk factors. In this nationwide study, increased age, interherd contact, breed, abortion management practices, and tick infestation density were identified as major risk factors for seropositivity, whereas co-herding with other animal species and a history of abortion were identified as protective factors.

### Limited role of tick infestation in Q fever seropositivity

Contrary to studies from the Middle East and North Africa reporting higher *C. burnetii* infection with increased tick density [22, 23, 37, 39], lighter tick infestation in this study was associated with higher sero-positivity. This counterintuitive result challenges the assumption of a direct role of ticks under the cross-sectional conditions of this study. Instead, the findings align with evidence from the European Union, where aerosol transmission is considered the primary route and ticks are not regarded as a major vector [40]. Supporting this, other studies have reported no significant association between tick infestation and seroprevalence [41], and a recent review concluded that tick-borne transmission is not the main route in livestock [42]. Nevertheless, these findings should be interpreted cautiously, as widespread acaricide use and temporal dynamics of infestation may obscure true associations. Overall, environmental and husbandry-related factors appear to play a more prominent role than tick burden in Q fever epidemiology under the current study design, warranting longitudinal investigations.

### Age as a consistent risk factor for seropositivity

The positive association between camel age (≥3 years) and *C. burnetii* seropositivity observed in this study is consistent with regional reports in camels [23, 37, 43] and findings in diverse ruminant species worldwide [33]. Older animals may act as key reservoirs within herds, contributing to sustained environmental contamination through prolonged exposure and age-related changes in immune efficiency. These findings suggest ongoing environmental shedding during parturition or other physiological events, reinforcing the need for targeted mitigation strategies such as segregated calving areas and focused monitoring of older animals.

### Co-herding with small ruminants and reduced seropositivity

Unexpectedly, camels co-herded with small ruminants exhibited lower seropositivity, contrasting with previous studies that identified co-herding as a risk factor [19, 23]. Given the established role of small ruminants, particularly goats, as high-shedding reservoirs [13, 18, 44], this inverse association may reflect genotype tropism, ecological segregation, or management practices limiting direct exposure. These findings suggest that the epidemiological risk of co-herding is mediated by local strain dynamics, interspecies interactions, and husbandry-specific exposure pathways.

### Interherd contact as a primary driver of infection

Consistent with prior epidemiological evidence [22, 44], contact with other camel herds emerged as a strong risk factor for seropositivity, supporting horizontal transmission via aerosolized infectious material from birth products, urine, or feces [45]. This reinforces the importance of interherd movement and mixing as critical drivers of transmission. Future molecular epidemiological studies, including genotyping of *C. burnetii* isolates from camels, small ruminants, and environmental samples, are essential to clarify strain circulation and host-pathogen dynamics.

### Novel association between coat color and seropositivity

This study identified a novel association between camel coat color and Q fever seropositivity, with brown-coated camels (Sofor) having higher odds of infection than lighter-coated camels (Waddaha). To our knowledge, this is the first epidemiological report linking coat color to *C. burnetii* infection in camels. Similar observations have recently been reported for *Anaplasma* infection in brown-coated camels in the United Arab Emirates [46]. Although speculative, genetic, physiological, or behavioral factors may underlie this association. Given the cross-sectional design, causality cannot be inferred, and validation in other populations is required.

### Inadequate disposal of aborted materials as a key driver

Disposal of aborted materials by burial or burning was associated with markedly higher odds of seropositivity compared with disposal in designated waste containers. This finding likely reflects inadequate implementation rather than the disposal principle itself. Shallow burial or open burning may fail to inactivate *C. burnetii*, a highly resistant pathogen that can persist in the environment for up to 24 months [42, 47]. Environmental contamination of soil, pasture, and water, coupled with aerosolization of contaminated dust, facilitates long-distance transmission [42, 48]. These results indicate that improper disposal practices are major drivers of environmental contamination and herd infection, necessitating urgent improvements in on-farm biosecurity and waste management.

### Inconsistent association between seropositivity and abortion history

Although *C. burnetii* is recognized as a cause of abortion, studies in camels have reported inconsistent associations between seropositivity and abortion history [4, 18, 22, 23, 37, 39, 41, 49]. In this study, herds with a history of abortion were 83% less likely to be seropositive, suggesting that *C. burnetii* may not be the primary driver of abortion in this population. Possible explanations include chronic infection with intermittent shedding, waning antibody titers, or management-related confounding. A negative serological association does not exclude *C. burnetii* as an etiological agent, as outbreaks have been attributed to the pathogen despite negative PCR results [50]. Longitudinal studies and direct detection of *C. burnetii* DNA in placental or fetal tissues are required to clarify causality.

### Spatial heterogeneity and regional implications

Marked spatial heterogeneity in seroprevalence was observed across governorates, ranging from 38.61% in Zarqa to 98.74% in Al-Tafilah, consistent with reports from Saudi Arabia [13] and Pakistan [22]. Higher prevalence in southern governorates may be driven by aridity, livestock density, low rainfall, and wind activity, conditions known to enhance aerosolization and dispersal of *C. burnetii* [11]. Conversely, lower prevalence in more urbanized areas likely reflects reduced camel density and limited exposure pathways. These findings underscore the need for region-specific surveillance and control strategies and highlight the potential role of cross-border strain circulation.

### High national burden and One Health implications

The pronounced discrepancy between molecular (68.93%) and serological (44.02%) prevalence suggests widespread active shedding alongside historical exposure. This may reflect early infection prior to seroconversion or detection of genetically related Coxiella-like organisms by PCR [40]. These findings highlight the limitations of using a single diagnostic approach and emphasize the value of combined serological and molecular testing. Using parallel interpretation, the weighted prevalence was 88.75% (95% CI: 79.26%–98.23%), one of the highest reported in the region. Collectively, these data confirm that camel herds in Jordan constitute a major reservoir for Q fever. The significant zoonotic risk necessitates urgent, coordinated interventions grounded in a One Health framework, integrating human, animal, and environmental health sectors to effectively control this endemic zoonosis.

## CONCLUSION

This nationwide, survey-weighted investigation provides robust evidence of a very high burden of Q fever in camel herds in Jordan. Using a combined diagnostic approach, the weighted prevalence based on parallel interpretation of ELISA and PCR reached 88.75%, indicating extensive environmental contamination and widespread circulation of *C. burnetii*. The substantial discrepancy between molecular (68.93%) and serological (44.02%) prevalence highlights active infection and shedding alongside historical exposure. Risk factor analysis identified increased age (≥3 years), interherd contact, Sofor coat type, inappropriate abortion material disposal, and tick infestation density as major drivers of seropositivity, whereas co-herding with other species and a history of abortion were associated with reduced odds of seropositivity. Marked spatial heterogeneity was observed, with southern governorates exhibiting the highest prevalence.

These findings confirm that camels represent a major reservoir for Q fever in Jordan and pose a significant zoonotic risk. Practical control measures should prioritize improving on-farm biosecurity, particularly by ensuring safe and effective disposal of aborted materials, regulating animal movement and interherd contact, and targeted monitoring of older animals. The limited epidemiological role of ticks under field conditions suggests that control efforts should focus more on environmental contamination, husbandry practices, and aerosol exposure pathways rather than vector-centric interventions alone. Integrating camels into national Q fever surveillance programs is essential to reduce transmission to humans, especially among high-risk occupational groups.

Key strengths include nationwide coverage, a representative cluster sampling design, the application of survey-weighted statistical methods, and the combined use of serological and molecular diagnostics. The integration of spatial analysis and comprehensive risk factor modeling provides a detailed epidemiological framework that improves generalizability and supports evidence-based decision-making within a One Health context.

The cross-sectional design limits causal inference and temporal interpretation of infection dynamics. Reliance on blood samples precluded direct assessment of active shedding through milk, vaginal secretions, or placental tissues. The exclusion of young camels may have influenced prevalence estimates, and widespread acaricide use may have obscured true associations with tick infestation. In addition, PCR-based detection may have included Coxiella-like organisms, potentially affecting molecular prevalence estimates.

Future studies should adopt longitudinal designs to clarify infection dynamics, shedding patterns, and the role of abortion events. Molecular characterization and genotyping of *C. burnetii* from camels, other livestock, humans, and environmental samples are needed to elucidate strain circulation, host specificity, and cross-border transmission. Further investigation into the biological basis of coat-related susceptibility and the inconsistent association with abortion is also warranted.

Overall, this study demonstrates that Q fever is hyperendemic in camel populations in Jordan, with camels functioning as a critical reservoir for *C. burnetii*. The magnitude of infection and associated zoonotic risks necessitate urgent, coordinated control strategies grounded in a One Health framework, linking animal, human, and environmental health to effectively mitigate this neglected but significant zoonosis.

## DATA AVAILABILITY

The supplementary data can be made available from the corresponding author upon request.

## AUTHORS’ CONTRIBUTIONS

RA: Conceptualization, methodology, study design, laboratory testing (serology/PCR), resources, data curation, and writing, review, and editing of the manuscript. MH: Conceptualization, methodology, investigation (field work/sampling), and manuscript revision. SB: Conceptualization, methodology, and study design. NR: Data analysis using the R programming language, data curation, and writing, review, and editing of the manuscript. All authors have read and approved the final version of the manuscript.
